# An Extrafollicular Adenomatoid Odontogenic Tumor Mimicking a Periapical Cyst

**DOI:** 10.1155/2018/6987050

**Published:** 2018-01-01

**Authors:** Farzaneh Mosavat, Roxana Rashtchian, Negar Zeini, Daryoush Goodarzi Pour, Shabnam Mohammed Charlie, Nazanin Mahdavi

**Affiliations:** ^1^Oral and Maxillofacial Radiology Department, School of Dentistry, Tehran University of Medical Sciences, Tehran, Iran; ^2^Oral and Maxillofacial Pathology Department, School of Dentistry, Tehran University of Medical Sciences, Tehran, Iran

## Abstract

Adenomatoid odontogenic tumor (AOT) is a rare noninvasive odontogenic tumor that occurs mostly in the second decade of life. Based on its tooth association, AOT can be classified into three categories of follicular, extrafollicular, and peripheral types; the follicular classification is considered as the most common type of AOT. This study reported a large extrafollicular case of AOT in a 40-year-old female. She was asymptomatic and tumor was detected accidentally by her dental practitioner. Since the panoramic radiograph showed a well-defined unilocular radiolucent lesion, we observed radiopaque spots within the lesion by using cone beam computed tomography. The extrafollicular type can mimic a periapical radiolucent lesion.

## 1. Introduction

Adenomatoid odontogenic tumor (AOT) is a slow-growing, well-defined tumor accounting for 3–7% of all odontogenic tumors [1]. Some authors consider AOTs to be benign and noninvasive neoplasms; however others describe them as developmental hamartomas odontogenic growths [2]. Although the AOT is considered as a low occurrence tumor in the literature, Philipsen et al. reported that AOT ranks fourth among the odontogenic tumors. The increasing number of reports in literature on AOT shows that the tumor develops more frequently than expected [3–5]. Depending on its location and tooth association, AOT can be divided into three classifications of follicular, extrafollicular, and peripheral type. About 70% of AOTs were identified as follicular, which is associated with an impacted permanent or supernumerary tooth; radiographic examination showed a well-circumscribed, unilocular radiolucent lesion which is diagnosed earlier in life than extrafollicular type (mean age of 17 years) [6–8].

The extrafollicular type is a central lesion that is not related to the embedded teeth, and the peripheral type is attached to the gingival structures [9]. Internal radiopaque focus was considered as one of the significant features of AOT, which can help its differential diagnosis from other bone cystic lesions [10]. Philipsen and Reichart showed that nearly two-thirds of AOTs had radiopaque spots inside the lesion [11]. The differential diagnosis of AOT from other lesions similar to AOT (e.g., dentigerous cyst, keratocyst odontogenic tumors, unicystic ameloblastoma, and calcifying cystic odontogenic tumors) in radiographic findings may be difficult. The ability of radiographic modality on showing the radiopaque foci within a lesion is essential for the diagnosis of AOT [7]. In the case of small opacification or superimposed area in the anterior region, CBCT is beneficial modality in demonstrating the detailed internal structures of lesions including radiopaque calcified spots [10].

## 2. Case Report

A 40-year-old female patient visited the Department of Oral and Maxillofacial Radiology of Tehran Dental School.

She was asymptomatic and the lesion was detected incidentally at routine radiography by her dental practitioner.

**Figure 1:**
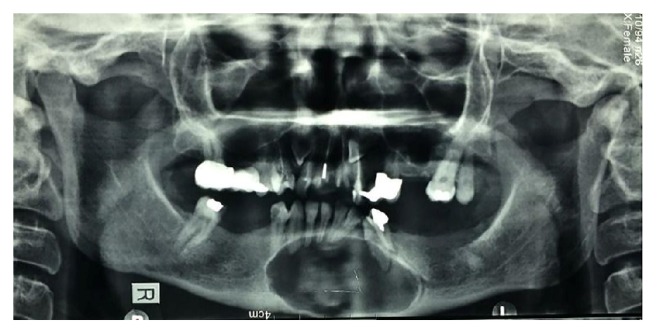
Panoramic radiograph shows a single large radiolucent lesion with well-defined border.

**Figure 2:**
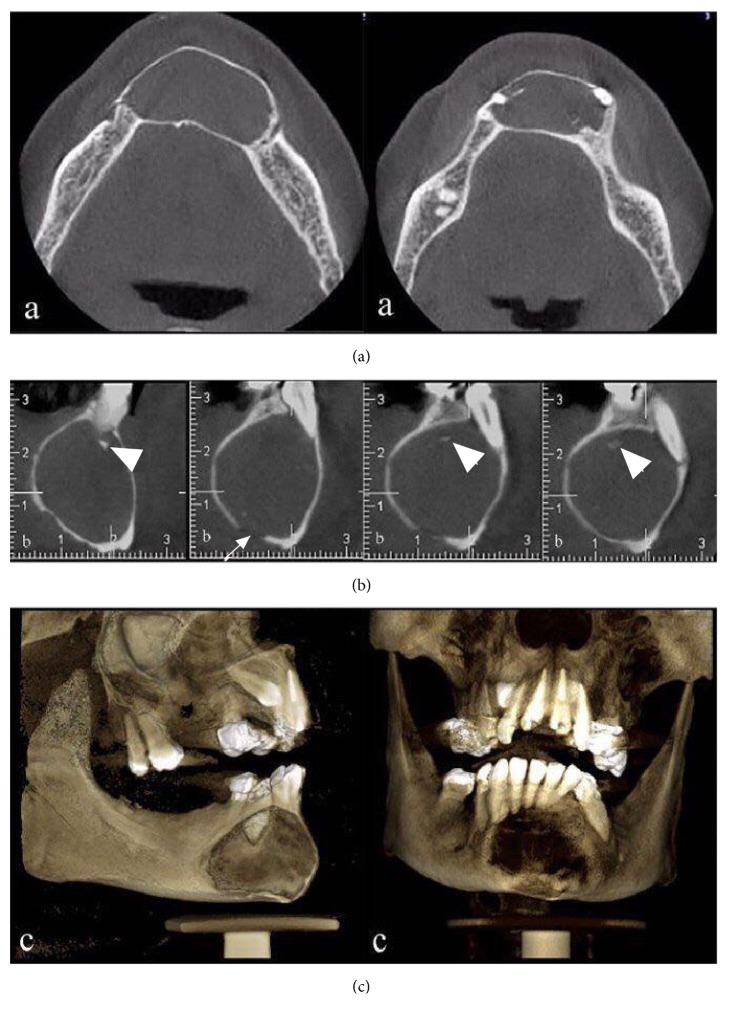
(a) Axial sections show that mental foramen is not involved but has close contact with border of the lesion at the left side. (b) Cross-sectional CBCT images reveal radiopaque spots inside the lesion indicated by white arrows in the image. (c) Three-dimensional volumetric surface rendering.

**Figure 3:**
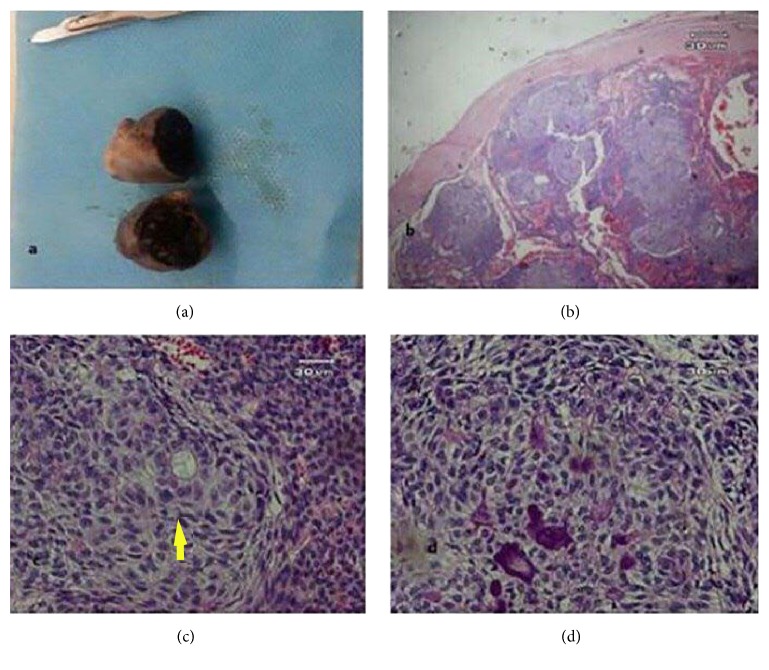
(a) On gross examination the lesion appears as an elliptical tissue, with 3.5 × 2.7 cm diameter. Cut section reveals a solid mass with multiple cystic spaces. (b) Low power view demonstrating a thick capsule surrounding the tumor (×40). (c) Duct-like structures which are the characteristic feature of AOT indicated by yellow arrow (400). (d) Spindle shaped cells that form whorled masses and rosette-like structures are noticeable (×400).

Intraorally, the patient had mild bony hard swelling in the anterior region of the mandible. The overlying mucosa was normal, and there was no sign of acute dentoalveolar or mucosal infection in the mandible region. The anterior mandibular teeth were displaced without mobility. The panoramic radiograph revealed a well-defined unilocular radiolucency with corticated rim, which extended from right to left mental foramens. Because of the lesion, the roots of the left lateral mandibular incisor and canine were deviated and resorbed (Figures 1 and 2(c)). The shadow of cervical spine was superimposed over the central part of the lesion (Figure 2(b)). Axial slice showed expansion of buccal and lingual cortical plates in the anterior mandible with perforation along the outer cortical plate at the left side (Figure 2(a)). Differential diagnosis included calcifying odontogenic cyst, central giant cell granuloma, AOT, and ameloblastoma. The lesion was completely enucleated. Microscopically, epithelial cells were arranged as spindle shaped cells in sheets and trabecular pattern and can form duct-like and rosette-like structures in a scant hyalinized stroma (Figure 3(d)).

On gross examination the lesion appears as an elliptical tissue, measuring about 3.5 × 2.7 cm in size (Figures 3(a) and 3(b)). Cut section of the mass revealed multiple cystic spaces and solid area. Small calcifications foci are scattered throughout the tumor. Small islands of tumoral cells have infiltrated the fibrous capsule (Figure 3(c)). Thus, the final diagnosis was given as extrafollicular AOT.

## 3. Discussion

AOT is a rare odontogenic tumor [12]. The prevalence of AOT is less than odontoma, cementoma, myxoma, and ameloblastoma [13]. AOT is a noninvasive, benign lesion representing 2–7% of all odontogenic tumors [14]. AOT usually appears in the age group of 5–50 years; two-thirds of the cases are diagnosed in the second decade of life, with an average age of 16 years. There is a predilection of AOT in females (female to male ratio = 1.9 : 1). At least 75% of lesions occur in the anterior maxilla, followed by the anterior mandible, and radiopacities were developed inside 77% of radiolucent lesions [2, 15]. As mentioned above, this tumor has two variants, that is, central and peripheral type (3% of all cases) [2, 16]. The peripheral type can be similar to a gingival fibroma or epulis [17]. Central tumor may have two types: (1) follicular type is associated with an impacted tooth (73% of all cases) and is often detected in mean age of 17 years and (2) extrafollicular type is often detected in mean age of 24 years (24% of all cases) [2, 3]. The extrafollicular type may appear as a periapical radiolucent lesion mimicking periapical cyst or intrabony defect [18, 19].

Radiographically, central AOT presents as well-defined, almost always unilocular radiolucency [20]. Expansion of the cortical plate can be presented. As a result of tumor expansion, adjacent teeth may be displaced. Tooth displacement is more common than root resorption [21]. This case had unusual radiographic features; it was huge extrafollicular AOT without any radiopaque foci in panoramic radiograph mimicking a periapical lesion. Although AOT occurs most often in second decade, the patient was a 40-year-old female. Late diagnosis of the present case could be due to slow growth and lack of interaction with tooth eruption. The most common site of extrafollicular AOT is anterior region of maxilla (incisor to canine). Our case was observed in the anterior region of mandible, which is the second common site [22]. It has reported that only 28% of AOT lesions occurred in the mandibular incisor area [23]. Generally in patient with AOT lesion, the lamina dura is commonly intact and periodontal ligament is normal. But, in our case, lamina dura cannot be radiographically detected and there was significant root resorption of the involved teeth. The lack of intact periodontal ligament and lamina dura in the involved teeth makes a more likely diagnosis of radicular cyst [18]. Since root resorption rarely occurred in AOT lesion, we detected displacement of the adjacent teeth (especially at the right side) and root resorption of the involved teeth [2]. The size of the current lesion was 3.5 × 2.7 cm; this was is consistent with the size of tumor used in the previous study, which was 1.5–3 cm in diameter [24]. Yilmaz et al. described an AOT causing painless swelling in the anterior mandible which was bony hard with no previous history of trauma, tenderness, discharge, or any other symptoms. These findings were consistent with that of our case [9]. CBCT has the superiority over panoramic radiograph in providing information on the detailed internal structure of the lesion; this can be ascribed to the small calcified area in the lesion. CBCT is the preferred option due to elimination of superimposition and high contrast resolution for mineralized tissue such as bones and calcified foci. Therefore, every single detail of a lesion is well depicted on CBCT images.

In summary, some clinical and radiographic features including age and radiolucent appearance of the lesion in a panoramic radiograph did not resemble AOT. However, CBCT assessment, due to its ability to provide more information from the internal structure of the lesion, suggests a differential diagnosis of AOT. Conservation surgical excision, with reoccurrence rate of 0.2%, is today's standard treatment. Some authors have reported that even incompletely removed lesion does not recur [17].

## 4. Conclusion 

The present case was described as an extrafollicular AOT mimicking a periapical lesion in a panoramic radiograph. In the case of small opacification or superimposed area in the anterior region, CBCT is beneficial modality in demonstrating the detailed internal structures of lesions including radiopaque calcified spots.
